# ESG performance and stock prices: evidence from the COVID-19 outbreak in China

**DOI:** 10.1057/s41599-022-01259-5

**Published:** 2022-07-18

**Authors:** Zengfu Li, Liuhua Feng, Zheng Pan, Hafiz M. Sohail

**Affiliations:** 1 School of Economics & Management, South China Normal University, Guangzhou, Guangdong China; 2 College of Management, Zhongkai University of Agriculture and Engineering, Guangzhou, Guangdong China

**Keywords:** Finance, Social science

## Abstract

This paper investigates the role of environmental, social, and governance (ESG) performance in stock prices during the market financial crisis caused by the COVID-19 pandemic. We use the Chinese listed company data as the bases for adopting an event-study method to identify the impact of ESG performance on cumulative abnormal returns. Empirical results suggest that ESG performance significantly increases firms’ cumulative abnormal returns and has asymmetric effects during the pandemic. Our results are robust to various robustness checks that consider the replacement of event window period, ESG measurement, adding other control variables, and sample exclusion of Hubei Province. We further find that reputation and insurance effects are important mechanisms through which ESG performance influences stock prices. Lastly, heterogeneous analyses show that ESG effects are considerably pronounced among firms with low human capital and bad image and in high-impact regions.

## Introduction

In recent years, environmental, social, and governance (ESG) investments, frequently called ethical or sustainable investments, have rapidly increased globally (Galbreath, 2013). ESG investing is an investment process that integrates ESG considerations into investment decisions (Mǎnescu, 2011). Given the COVID-19 pandemic, the necessity of ESG investing has been highlighted again (Demers et al., 2021; Manabe and Nakagawa, 2022). Investors are interested in ESG investments for at least two reasons (Renneboog et al., 2008). First, by focusing on ESG investments, ethical investment practices are actively promoted (Baldini et al., 2018; Broadstock et al., 2021). Second, ESG investments are increasingly recognized as improving the performance of managed portfolios, reducing portfolio risks, and increasing returns (Albuquerque et al., 2020; Díaz et al., 2021; Broadstock et al., 2021).

Early literature on ESG investing has been partially inspired by studies of the eminent economist Milton Friedman (Friedman, 2007), who argued that ESG practices constitute a misallocation and misappropriation of valuable corporate resources. Renneboog et al. (2008) concluded that existing studies hint, but do not explicitly demonstrate, that ethical investors are willing to accept sub-optimal financial performance to pursue social or ethical objectives. Subsequently, a series of studies have expanded on the preceding literature. Some studies on ESG investing have focused on the application of returns and risk management. Hartzmark and Sussman (2019) found that investors make positive predictions on sustainable assets, steering money away from funds with low portfolio sustainability ratings to those with high ratings. They also found no evidence that high-sustainability funds outperform low-sustainability funds. Demers et al. (2021) determined that ESG performance facilitates the accumulation of intangible assets but does not serve as protection against downside risk.

However, emerging studies have supported the view that ESG-themed investments have low downside risks and are minimally volatile in price during turbulent times. Hoepner et al. (2021) and Pedersen et al. (2021) obtained empirical evidence that ESG engagement reduces firms’ downside risks and their exposure to downside risk factors. Albuquerque et al. (2020) developed a theoretical framework to show that stocks with high ESG ratings have significantly higher returns, lower return volatilities, and higher trading volumes than other stocks. Broadstock et al. (2021) showed that high-ESG portfolios typically outperform low-ESG portfolios, thereby mitigating financial risks during financial crises.

Although there is limited research on the specific role of ESG performance during times of crisis, some insights have been gained from the 2008–2009 global financial crisis. Nejati et al. (2010) noted that the root causes of the current economic crisis could be moderated by a global transparency and accountability system and a public reporting of ESG performance. Erragraguy and Revelli (2015) showed that the adoption of ESG standards by firms during the crisis increased transparency, mitigated information asymmetries, and improved stock market liquidity and quality. Henke (2016) demonstrated that high-ESG-rated funds outperformed low-ESG-rated funds during the crisis, further supporting the view that investors place intrinsic value on ESG investments.

In the first few months of 2020, the sudden market-wide financial crisis was triggered in response to the emerging global health crisis (i.e., COVID-19), the consequences of which were more severe than those of the Great Depression in 1929–1933 and the global financial crisis in 2007/2008 (Broadstock et al., 2021). The current study shows that stock prices are empirically tested for negative shocks during the COVID-19 pandemic in some products and firms but not in others (Al-Awadhi et al., 2020; Shen et al., 2020). This result leads to a compromise opinion that the role of ESG generally depends on exposure to crisis shocks. Moreover, we are motivated to question how ESG effects vary along with different products and firms during public crises. However, only a few studies have indicated the specific role of ESG performance in crisis periods. Therefore, the goal of this paper is to fill in this research gap.

We use the Chinese listed company data as the bases for adopting an event-study method to identify the impact of ESG performance on cumulative abnormal returns. The empirical results show that ESG performance is positively associated with cumulative abnormal returns during the COVID-19 pandemic. When decomposing firms with positive and negative shocks, we find that cumulative abnormal returns are positively related to ESG among firms with negative shocks but not positive shocks. These results suggest that the importance of ESG performance is reinforced in times of crisis, and is consistent with the inference that investors use ESG performance as a signal of future returns and risk mitigation.

Our work builds on the current literature on the role of ESG performance in stock prices (Duuren et al., 2016; Remmer Sassen et al., 2016; Jagannathan et al., 2017) and extends the results on ESG to public crisis events. We use an event study approach to analyze the volatility of ESG performance on stock prices during the COVID-19 pandemic. Compared with the existing literature (Nejati et al., 2010; Erragraguy and Revelli, 2015; Henke, 2016), this study provides a more comprehensive perspective on the importance of ESG performance in major crises. Understanding the importance of ESG is necessary because it is a crucial indicator of risk management, non-financial performance, and sustainability. Through ESG practices, firms can obtain significant reputation and risk protection to reduce price volatility in times of crisis, thereby contributing to their long-term operations and sustainability.

This study adds to the limited number of prior studies that have examined the impact of firms’ ESG performance on their stock prices but have provided conflicting results (Friedman, 2007; Renneboog et al., 2008; Hartzmark and Sussman, 2019; Demers et al., 2021). The current study also provides additional empirical evidence on the important mechanisms of ESG performance through further analysis, thereby opening a black box for a positive relationship between ESG performance and stock prices. The results of this study suggest that reputation and insurance effects are important mechanisms by which ESG performance influences stock prices. This outcome reflects the fact that sustainability investments have low downside risks and are minimally volatile in price during turbulent periods. This finding clarifies the important role of current ESG practices in guiding investors’ decision-making and provides empirical evidence for investors to focus on sustainable investment.

This paper also complements the literature on the effects of public crises on financial markets. Existing literature has discussed the influence on financial markets mainly in terms of the 2008–2009 financial crisis (Nejati et al., 2010; Erragraguy and Revelli, 2015; Henke, 2016). We differ from these studies by specifically focusing on the COVID-19 pandemic. Note that the subject of our study is Chinese listed companies. Given that China is the second-largest economy in the world where the COVID-19 public crisis spread earlier and was interrupted by containment measures, a reasonable undertaking is to investigate the influence of this crisis on financial markets in the Chinese sample we use compared with other countries.

The remainder of this paper is organized as follows. Section “Data and methodology” presents the sample and variables. Section “Empirical results” discusses the main empirical results and robustness analyses. Section “Further analysis” provides further mechanisms. Lastly, section “Conclusion” concludes the study.

## Data and methodology

### Data

Data of this study cover Chinese non-financial A-share listed firms in 2020. We collect data from several resources. First, we acquire ESG data from China Sino-Securities Index Information Service (Shanghai) Company Limited, a third-party data provider based in China specializing in ESG data. Second, stock prices, firm financial data, and firm management data are obtained from China Securities Markets and Accounting Research Database. In particular, stock prices are measured using cumulative abnormal returns calculated by utilizing the event-study method. Third, we obtain media coverage data mainly from the Chinese Research Data Service database to measure the level of media attention.

### Main variables

Data of this study cover Chinese non-financial A-share listed firms in 2020. We collect data from several resources. First, we acquire ESG data from China Sino-Securities Index Information Service (Shanghai) Company Limited, a third-party data provider based in China specializing in ESG data. Second, stock prices, firm financial data, and firm management data are obtained from the China Securities Markets and Accounting Research Database. Specifically, stock prices are measured using cumulative abnormal returns calculated by the event-study method. Third, we obtain media coverage data mainly from the Chinese Research Data Service database to measure the level of media attention.

#### Stock price (CAR)

Cumulative abnormal return (CAR) is widely used as a measure of stock price. We follow the previous literature (Demers et al., 2021; Zhang et al., 2021; Broadstock et al., 2021) and use CAR estimated through an event study approach as the dependent variable. CAR is an impartial estimate of additional or reduced firm value that accrues as a result of the occurrence (McWilliams and Siegel, 1997; Campbell et al., 1998; Fernando et al., 2012). In the COVID-19 context, this paper uses the event study method to analyze the impact of ESG performance on stock prices.

In an event study, we need to determine the event date, event window, estimation window, and estimation model. The details are as follows. (1) *Event date*: As COVID-19 received national attention on 20 January 2020, we draw on existing literature (Zhang et al., 2021) and use this date as an event date in the event study. On 20 January 2020, China’s central government provided important instructions on the prevention and containment of COVID-19. On the same day, panic ensued after the announcement by pulmonary experts that COVID-19 was more transmissible than previous diseases and could be passed from one person to another person. As of 20 January 2020, the Chinese National Health Council officially issued the number of new cases in each province and incorporated COVID-19 into the Infectious Diseases Act and Sanitary and Isolation Act (Zhang et al., 2021). (2) *Event window*: This paper follows previous studies (Kanas, 2005; Miyajima and Yafeh, 2007; Fernando et al., 2012) and selects the event window consisting of 11 days from *t*_−5_ to *t*_+5_ days. (3) *Estimation window*: Campbell et al. (1998) documented that the selection of estimation window for short-term events can be 120 days or even longer. Thus, we choose the estimated window period of 175 days from *t*_−210_ to *t*_−36_ days. (4) *Estimation model*: We use the existing literature (Campbell et al., 1998) as a basis to take the OLS market model for calculating the expected returns.

This study requires the calculation of expected, abnormal, and cumulative abnormal returns (Campbell et al., 1998). We first calculate each firm’s expected return (ER_*i,t*_) during the event period:

1 $${\rm {ER}}_{i,t} = \beta _0 + \beta _1R_{M,t} + \varepsilon ,$$

where ER_*i,t*_ is the expected return of firm *i* on day *t* during the event period, *R*_*M*,*t*_ represents the market return on day *t* during the event period, and *β*_0_ and *β*_0_ are the estimated parameters in Model (1). Thereafter, we use expected returns (ER_*i*,*t*_) gained from Model (1) to calculate the abnormal returns during the event period. This study estimates abnormal returns (AR_*i,t*_) based on the Fama et al. (1969) market model:

2 $${\rm {AR}}_{i,t} = R_{i,t} - {\rm {ER}}_{i,t},$$

where AR_*i,t*_ is the abnormal return of firm *i* on day *t* during the event period, *R*_*i,t*_ is the actual return of firm *i* on day *t* during the event period, and ER_*i*,*t*_ is the expected return obtained from Model (1). Lastly, we use abnormal returns (AR_*i,t*_) obtained from Model (2) to calculate the cumulative abnormal returns:

3 $${\rm {CAR}}_{i,t} = \mathop {\sum}\limits_k^j {{\rm {AR}}_{i,t}} ,$$

where CAR_*i,t*_ is a calculation from the period of days between *k* and *j*. In this paper, the event windows to calculate CAR is [−5, 5].

#### ESG performance (ESG)

The Independent variable used in this study is the quarterly score of ESG performance, which is calculated based on three dimensions: environmental, social, and governance. Given that ESG data are disclosed quarterly, we calculate the average of ESG over four quarters to measure the annual ESG performance. We also follow the previous literature (Zhang et al., 2021; Broadstock et al., 2021; Díaz et al., 2021; Demers et al., 2021) and include the following set of control variables in the estimation: leverage ratio (Lev), firm profitability (Roa), firm size (Size), nature of equity (Soe), degree of risk (Beta), shareholder structure (Top1), number of board members (Board), the duality of CEO and chairman (Dual), the proportion of independent directors (Independ), intangible asset (Intangible), and tangible asset (Tangible). The detailed definitions of the variables are presented in Table 1.

**Table 1:** Variable definitions.

Variable	Description	Definition
CAR	CAR[−5, 5]	The cumulative abnormal return for the five days before and after the event date
ESG	ESG performance	ESG disclosure score of the WSCI for the year before the event date divided by 100
Lev	Leverage	Ratio of total liabilities to total assets
Roa	Profitability	Ratio of net earnings to total gross sales
Size	Firm size	Natural logarithm of total assets
Soe	Nature of equity	If a firm is state owned, the value is 1, and 0 otherwise
Bate	Degree of risk (CAPM Beta)	Factor loading on the market return from a Fama–French and Carhart four-factor model of daily returns over the trading days prior to the year before the event date.
Top1	Ownership structure	Ratio of the largest shareholder
Board	Board size	Natural logarithm of the number of directors
Dual	Board leadership	A dummy variable of one if the CEO is the chair of the board
Independ	Board independence	The proportion of outside independent directors
Intangible	Intangible asset	Ratio of intangible assets to total assets
Tangible	Tangible asset	Ratio of tangible assets to total assets

### Summary statistics

In response to the COVID-19 pandemic, this paper selects Chinese A-share listed companies as a study sample. This study selects the sample based on the following considerations. First, the financial sector is excluded owing to the uniqueness of its business, financial reporting, and regulatory structure. Second, we exclude firms with losses and those specially treated by stock exchanges. Third, we exclude samples with an estimation window of under 175 trading days. Fourth, our article removes samples with missing values in the ESG, CAR, and control variables. Lastly, continuous variables are winsorized at the 1% and 99% levels to mitigate concerns with extreme values. The resulting sample in our study is 2188 observations.

Table 2 provides the descriptive statistics of the variables. CAR index ranges from −0.2496 to 0.4665. In addition, the mean and median of CAR are −0.0115 and −0.0367, respectively, with a standard deviation of 0.1223. This result suggests that the level of cumulative abnormal returns varies considerably across firms and that overall cumulative abnormal returns are low. Moreover, this situation implies that firms are generally subject to negative shocks during the COVID-19 pandemic. The average ESG is 0.8447, which is within the range of good. For the control variables, the average leverage is 44.21%, ROA is 5.13%, and firm size is 22.59. Our sampled chairman has ~27.06% of the CEO, and the largest shareholder holder accounts for 34.61% of firm stocks. The average board size is about 8 (=*e*^2.1260^) members, 37.61% of whom are independent directors. This result is consistent with the CSRC requirement for board independence (Wang et al., 2021). The distribution of the control variables in this paper is similar to that reported in previous research (Zhang et al., 2021; Broadstock et al., 2021; Díaz et al., 2021).

**Table 2:** Descriptive statistics.

Variables	Mean	P50	Std	Min	Max	*N*
CAR	−0.0115	−0.0367	0.1223	−0.2496	0.4665	2188
ESG	0.8447	0.8413	0.0626	0.6824	0.9695	2188
Lev	0.4421	0.4332	0.1947	0.0714	0.8660	2188
Roa	0.0513	0.0694	0.1672	−0.9242	0.3348	2188
Size	22.5890	22.4286	1.3525	17.9544	28.1935	2188
Soe	0.3944	0.0000	0.4888	0.0000	1.0000	2188
Bate	1.1319	1.1328	0.2428	0.5230	1.7427	2188
Top1	34.6056	32.3150	14.7837	4.7600	81.1900	2188
Board	2.1260	2.1972	0.1983	1.6094	2.7081	2188
Dual	0.2706	0.0000	0.4444	0.0000	1.0000	2188
Independ	0.3761	0.3636	0.0539	0.3000	0.5714	2188
Intangible	0.0467	0.0347	0.0512	0.0000	0.3403	2188
Tangible	0.9247	0.9529	0.0858	0.5340	1.0000	2188

Table 2 presents the descriptive statistics for the main regression variables. All variables are winsorized at the 1st and 99th percentiles.

**Fig. 1:**
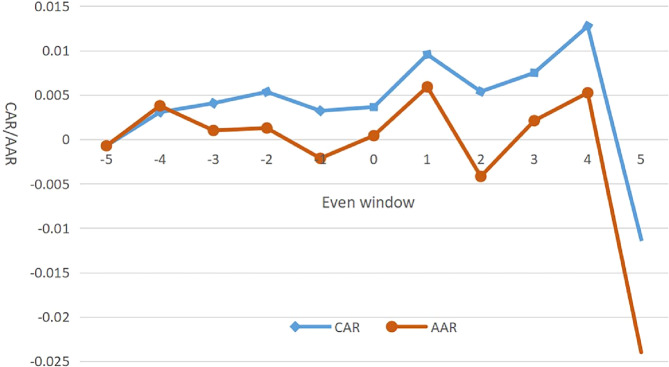
Average abnormal returns and cumulative abnormal returns trends. Trend of AARs and CARs for the event window (*t*_−5_–*t*_+5_ days).

Figure 1 shows a graphical representation of AARs and CARs for the event window [−5, 5]. As shown in Fig. 1, the two trend lines rise initially and fall thereafter from the announcement date. In the 4 days from 20 to 23 January 2020, firms reacted positively to the pandemic market. In the early stage of the COVID-19 pandemic, the demand for products to prevent pandemic infection was greater than the supply. This phenomenon prompted speculators to use arbitrage opportunities to invest, thereby explaining the positive response of the capital market.

On the 4th day of the COVID-19 outbreak (23 February 2020), the city of Wuhan, which was the most affected city, was closed. Shortly after the lockdown, the stock market was closed for the Chinese Spring Festival from 24 January to 2 February. The market reopened on 3 February. Note that on the 5th day of trading following the COVID-19 outbreak, which was the first trading day after the closure of Wuhan (3 February 2020), the response of firms to the pandemic was extremely negative. The pandemic caused chaos and weakened the economy of China, particularly after the closure of Wuhan. The closure of the city to curb the further spread of the disease will destroy the entire logistics and supply chain system (Tang et al., 2021). Most factories faced shutdowns, production stoppages, and even closures, thereby causing a significant downward trend across the capital markets. The downward trend of the stock market was consistent with the previous statistical description that the COVID-19 epidemic in 2019 has had a significant negative impact on the financial market.

## Empirical results

### Effect of ESG performance on firms’ stock price

We use the following multiple regression model to investigate the general relationship between ESG performance and firm stock prices during the COVID-19 pandemic:

4 $${{CAR}}_i = \beta _0 + \beta _1{{ESG}}_i + {{Controls}}_i + {{province}}\,{{FE}} + {{Industry}}\,{{FE}} + \varepsilon _i$$

where *i* denotes the firm, CAR represents the firm’s cumulative abnormal return during the COVID-19 pandemic, and *ESG* is the firm’s ESG performance measure proxied by its ESG score. Our results focus on *β*_1_, which captures how ESG performance affects a firm’s stock price. The vector Controls_*i*_ stacks a series of control variables that account for the impact of firm characteristics on the stock price. Details of these variables are described in the section “ESG performance (ESG)”. We also include province-fixed effects to control for unobservable regional characteristics. ESG is a highly industry-related variable (Yu and Luu, 2021). To control for industry characteristics, we control for industry fixed effects, in which *ε*_*i*_ is the error term. Variables and definitions used in the model are shown in Table 1.

We report the results of the baseline OLS regressions in Table 3. In column (1), we include only the ESG and CAR indicators after controlling for the province- and industry-fixed effects. The coefficient of ESG is 0.1479 (*t*-stat 3.5072), which is positive and statistically significant at the 1% level. This result indicates that ESG practices play an important role in reducing price volatility in times of crisis. Our results show that ESG performance has a significant positive impact on the cumulative excess return. In other cases, we include firm control variables in column (2). However, estimates remain positive and statistically significant. For control variables, we find that the coefficient of Size is significantly positive, suggesting that large firms reduce their risk of stock price declines. Conversely, the coefficient of Lev is negative, implying that firms with more leverages will suffer a greater risk of stock price declines. Our results are consistent with those of previous studies regarding control variables (Demers et al., 2021; Broadstock et al., 2021). In summary, our results provide suggestive evidence that firms with high ESG ratings are conducive to mitigating the downside risk of stock prices during the COVID-19 pandemic.

**Table 3:** Baseline results.

Variables	(1)	(2)
ESG	0.1479*** (3.5072)	0.1316*** (2.7766)
Lev		−0.0655*** (−4.0606)
Roa		0.0021 (0.1384)
Size		0.0143*** (5.9616)
Soe		−0.0025 (−0.3939)
Bate		0.0624*** (5.3495)
Top1		−0.0003* (−1.7543)
Board		0.0029 (0.1907)
Dual		0.0124* (1.9414)
Independ		0.0002 (0.0034)
Intangible		−0.0708 (−0.9197)
Tangible		−0.1300** (−2.3958)
Constant	−0.1365*** (−3.7895)	−0.3626*** (−4.4803)
Observations	2188	2188
*R*-squared	0.114	0.149
Province fixed effects	Yes	Yes
Industry fixed effects	Yes	Yes

This table presents the baseline results for the impact of ESG performance on the cumulative abnormal return. The dependent variable is CAR in columns (1) and (2). Detailed variable definitions are presented in Table 1. All variables are winsorized at the 1st and 99th percentiles. *t*-statistics are reported below coefficient estimates and are calculated based on robust standard errors. *, **, and *** denote significance at the 10%, 5%, and 1% levels, respectively.

To significantly understand whether or not the role of ESG depends on the risk exposure characteristics of crisis shock, this paper examines the influence of ESG performance on stock prices of firms subject to positive and negative shocks during the COVID-19 pandemic. We choose two approaches to classify the sample into firms with positive and negative shocks during the pandemic. On the one hand, this paper draws on the existing literature (Kanas, 2005; Miyajima and Yafeh, 2007) in classifying the positive and negative shock groups by determining whether or not the cumulative abnormal return is above 0. On the other hand, we use the previous literature (Al-Awadhi et al., 2020; Shen et al., 2020) as a basis for identifying the following industries as positive groups: information technology and medicine manufacturing industries. Moreover, we select the following industries to be identified as negative groups: tourism, transportation, restaurants, wholesale and retail trade, realty business, and export manufacturing industries.

Table 4 shows the results of the tests of exposure characteristics during the crisis. The first two columns and last two columns show that the results are similar whether the sample is divided in the first or second way. Coefficients of ESG for firms in the negative group are positive and statistically significant. Conversely, coefficients of ESG for firms in the positive group is not statistically significant. These results suggest that the positive effect of ESG performance on stock prices is more significant among firms that are more severely affected by negative shocks. That is, our findings provide evidence that ESG performance acts as a risk protection tool that contributes to the sustainability of operations in turbulent times, specifically among firms with severe negative shocks.

**Table 4:** Risk exposure test results.

Variables	(1)	(2)	(3)	(4)
	Cumulative excess return		Industry	
	High risk exposure	Low risk exposure	High risk exposure	Low risk exposure
ESG	−0.0550 (−0.5955)	0.0927*** (3.5646)	0.0387 (0.2201)	0.2195** (2.2060)
Constant	0.4465*** (2.9646)	−0.4716*** (−10.6212)	−0.1792 (−0.7021)	−0.3320** (−2.2043)
Observations	720	1,467	280	499
*R*-squared	0.113	0.185	0.317	0.147
Controls	Yes	Yes	Yes	Yes
Province fixed effects	Yes	Yes	Yes	Yes
Industry fixed effects	Yes	Yes	Yes	Yes

This table presents the baseline results for the impact of ESG performance on the cumulative abnormal return. The dependent variable is CAR in Table 4. We control for the Lev, Roa, Size, Soe, Bate, Top1, Board, Dual, Independ, Intangible, and Tangible. Detailed variable definitions are presented in Table 1. All variables are winsorized at the 1st and 99th percentiles. *t*-statistics are reported below coefficient estimates and are calculated based on robust standard errors. *, ** and *** denote significance at the 10%, 5%, and 1% levels, respectively.

### Robustness tests

#### Alternative event window periods

In the baseline regression, we mainly use an event window of [−5, 5] for the test. However, the role of ESG performance for the shorter or longer event windows is unclear. This section investigates whether or not our results hold after replacing the shorter and longer event windows. These event windows are as follows: [−3, 3], [−7, 10], [−7, 11], and [−7, 14]. The first four columns of Table 5 present the results of the tests for alternative event window periods, in which each column represents a different event window. Column (1) shows that the coefficient of ESG is 0.0752 (*t*-stat 2.8186) in the short-term event window [−3, 3], which is positive at the 1% level. We also find that the coefficient of ESG is 0.1327 (*t*-stat 2.6966) in the long-term event window [−7, 10], which is positive at the 1% level. Coefficient of ESG is also significantly positive in the event windows [−7, 11] and [−7, 14], albeit at a weaker level of significance (5%, 10%). In summary, these findings provide strong evidence that ESG plays an important role in preventing downside risk during turbulent times.

**Table 5:** Results of robustness tests.

Variables	(1)	(2)	(3)	(5)	(6)	(7)	(8)	(9)
	CAR[−3+3]	CAR[−7,+10]	CAR[−7,+11]	CAR[−7,+14]	CAR	CAR	CAR	CAR
ESG	0.0752*** (2.8186)	0.1327*** (2.6966)	0.1239** (2.4487)	0.0994* (1.9121)			0.1317*** (2.72)	0.1288*** (2.71)
ESG1					0.1322*** (2.8273)			
ESG2						0.1363*** (2.9299)		
Constant	−0.1260*** (−2.7609)	−0.1810** (−2.0266)	−0.1465 (−1.5726)	−0.1706* (−1.8982)	−0.3633*** (−4.4923)	−0.3660*** (−4.5283)	−0.3462*** (−4.21)	−0.3457*** (−4.14)
Observations	2188	2188	2188	2188	2188	2188	2136	2188
*R*-squared	0.121	0.138	0.139	0.136	0.149	0.150	0.151	0.150
Controls	Yes	Yes	Yes	Yes	Yes	Yes	Yes	Yes
Province fixed effects	Yes	Yes	Yes	Yes	Yes	Yes	Yes	Yes
Industry fixed effects	Yes	Yes	Yes	Yes	Yes	Yes	Yes	Yes

This table presents the robustness results for the impact of ESG performance on the cumulative abnormal return. We control for the Lev, Roa, Size, Soe, Bate, Top1, Board, Dual, Independ, Intangible, and Tangible. Detailed variable definitions are presented in Table 1. All variables are winsorized at the 1st and 99th percentiles. *t*-statistics are reported below coefficient estimates and are calculated based on robust standard errors. *, **, and *** denote significance at the 10%, 5%, and 1% levels, respectively.

#### Alternative proxies for ESG performances

Given that raw data for the ESG score is quarterly, this paper uses it for testing the annual data of firms that are susceptible to measurement error and raise concerns about their validity. To address these potential issues, we consider two alternative proxies for ESG performance. First, we choose the median of the quarterly data on ESG scores (ESG1) to measure ESG performance. Second, we measure ESG performance as ESG score in the first quarter of year *t* + 1 (ESG2). Similarly, we find a robust positive association between ESG performance and stock price, as shown in columns (5) and (6) of Table 5.

#### Excluding the Hubei sample

This paper considers a subsample that excludes Hubei Province, which was the most affected by COVID-19. The early outbreak of COVID-19 in China was concentrated in Wuhan (Wuhan), Hubei Province, and spread rapidly to other provinces. Hubei has many listed firms. Thus, we draw on the existing literature (Ren et al., 2021) and exclude the Hubei sample to emphasize that our results are not driven by firms in the province most affected by COVID-19. We re-estimate our regression using the subsamples. Column (7) of Table 5 shows that the coefficient of ESG is positive and significant, which remains consistent with our findings.

#### Inclusion of other variables

Existing research has suggested that institutional investor ownership and cash holdings of firms may be associated with stock price volatility (Bushee and Noe, 2000; Chang et al., 2017). Whether or not our regression results are sensitive to include institutional investor ownership and cash holdings has aroused our concern. To mitigate this concern, we control for institutional investor ownership and cash holdings of firms in the regression process. In column (8) of Table 5, empirical results remain positive and statistically significant for the cumulative abnormal returns. The inclusion of firms’ institutional investor ownership and cash holdings does not induce material change in coefficient magnitudes. That is, empirical evidence implies that the inclusion of institutional investor ownership and cash holdings should not be an issue in our research.

## Further analysis

### Potential mechanism

This section extends the preceding results to clarify the potential mechanisms of why ESG performance positively affects firms’ cumulative abnormal returns.

#### Reputation effect

Demers et al. (2021) suggested that ESG performance is an intangible asset, which plays a positive role in optimizing supply chain partnerships, enhancing consumer product satisfaction, and improving employee productivity. Given the attention of social media and stakeholders, ESG practices are gradually becoming a tool for corporate impression management that can enhance social visibility and expand market share (Lokuwaduge and Heenetigala, 2017; Xie et al., 2019). Thus, we expect ESG performance to be conducive to increasing cumulative abnormal corporate returns through reputation enhancement.

In particular, we take the number of positive online media and financial newspaper reports to represent the reputation gained by the firm. Thereafter, we use the natural logarithm of the number of positive online media and financial newspaper reports as our variables New1 and New2, respectively. To test the reputation effect mechanism, we introduce an interaction term between ESG and New1 or New2 in our model. As shown in columns (1) and (2) of Table 6, the coefficients of New1 and New2 are significantly positive, suggesting that ESG practices are beneficial in enhancing corporate reputation. We also find that the coefficients of ESG × New1 and ESG × New2 are significantly negative, implying that the reputation effect of ESG performance is more pronounced among firms with lower reputations. Our findings demonstrate that ESG practices are beneficial in enhancing corporate reputation to improve cumulative excess returns during turbulent times.

**Table 6:** Results of the mechanism test.

Variables	(1)	(2)	(3)	(4)
ESG	0.4907*** (2.9067)	0.3380*** (3.8429)	0.0105 (0.1739)	0.0247 (0.3991)
New1_ESG	−0.0903** (−2.2361)			
New1	0.0831** (2.3496)			
New2_ESG		−0.0961*** (−3.1402)		
New2		0.0867*** (3.2737)		
Risk1_ESG			0.7086** (2.4027)	
Risk1			−0.7054*** (−2.8392)	
Risk2_ESG				0.0675** (1.9851)
Risk2				−0.0671** (−2.3375)
Constant	−0.6460*** (−3.8372)	−0.4924*** (−4.5006)	−0.2335*** (−2.6378)	−0.2518*** (−2.8153)
Observations	2188	2188	2188	2188
*R*-squared	0.153	0.156	0.161	0.158
Controls	Yes	Yes	Yes	Yes
Province fixed effects	Yes	Yes	Yes	Yes
Industry fixed effects	Yes	Yes	Yes	Yes

This table presents the mechanism test results for the impact of ESG performance on the cumulative abnormal return. The dependent variable is CAR in Table 6. We control for the Lev, Roa, Size, Soe, Bate, Top1, Board, Dual, Independ, Intangible, and Tangible. Detailed variable definitions are presented in the Table 1. All variables are winsorised at the 1st and 99th percentiles. *t*-statistics are reported below coefficient estimates and are calculated based on robust standard errors. *, ** and *** denote significance at the 10%, 5%, and 1% level, respectively.

#### Insurance effect

Academics and practitioners agree that firms’ risk exposures are linked to their ESG profiles. Albuquerque et al. (2020) presented empirical evidence suggesting that firms’ increasing product differentiation through ESG investments reduces systemic risk and improves firm value. Hoepner et al. (2021) found corroborating evidence that ESG practices reduce firms’ exposure to downside risk factors. These results support practitioner arguments that including ESG factors in investment decisions can mitigate uncompensated portfolio risks (Jagannathan et al., 2017; Pandey and Kumari, 2021; Broadstock et al., 2021). Hence, we expect ESG performance to be considered a risk management tool during the COVID-19 pandemic to increase cumulative excess returns by reducing business risk.

This paper Utilizes the previous literature (Ghosh and Olsen, 2009) as basis in using the standard deviation of sales revenue over the past 5 years (Risk1) to measure business operating risk. To remove the effect of industry, we also use the standard deviation of sales revenue over the past 5 years adjusted for industry (Risk2) to measure business operating risk. We introduce an interaction term between ESG and EU1 or EU2 in the model to test the insurance effect mechanism. The results in columns (3) and (4) of Table 6 show that the coefficients of Risk1 and Risk2 are negative and significant. This result is consistent with findings of previous studies that ESG performance reduces downside risk. We also find that coefficients of ESG × Risk1 and ESG × Risk2 are significantly positive, suggesting that the insurance effect of ESG performance is markedly pronounced among firms with high operational risk. The results of this study indicate that ESG practices can be used as a risk management tool in turbulent times, thereby increasing cumulative abnormal returns. These findings further support the insurance-enhancing effects of ESG practices.

### Cross-sectional analysis

In this subsection, we conduct several cross-sectional tests to examine how the impact of ESG performance on cumulative abnormal returns varies with firm characteristics during the COVID-19 pandemic.

#### Cross-sectional tests of human capital characteristics

We investigate whether or not the human capital characteristics of firms affect the contribution of ESG performance in terms of cumulative abnormal returns during the COVID-19 pandemic. Apart from the detrimental influence of the virus on staff safety, the lockdown and physical separation measures damage firms’ financial performance. Fahlenbrach et al. (2020) showed that labor-intensive firms, in which work-from-home policies are difficult to implement, have high exposure to COVID-19. By contrast, firms with high technological equipment are less affected and even have the opportunity to expand their business (Li et al., 2021). Thus, we predict that firms with low human capital are considerably at risk during the COVID-19 pandemic, thereby possibly increasing the sensitivity of ESG performance to cumulative abnormal returns.

This paper draws on prior literature (Li et al., 2021) and selects the ratio of the number of employees to sales as a measure of human capital intensity (Labor). The more value of Labor, the higher the productivity of employees and the higher the human capital of the firm. In addition, we divide the two groups of high and low human capital according to whether or not Labor is above the median value of the sample. Columns (1) and (2) of Table 7 show that the coefficient of ESG is significantly positive at the 1% level among firms with low human capital, but it is not statistically significant among firms with high human capital. Thus, the role of ESG performance in preventing stock price downside is markedly significant among firms with low human capital during the COVID-19 pandemic. This result is consistent with our expectations.

**Table 7:** Results of heterogeneity test.

Variables	(1)	(2)	(3)	(4)	(5)	(6)
	Human capital		Negative report		Epidemic regions	
	Above Median	Below Median	Above Median	Below Median	Yes	No
ESG	0.0321 (0.4409)	0.2350*** (3.6715)	0.2104*** (3.3078)	0.0323 (0.4339)	0.1465** (2.4184)	0.0953 (1.2877)
Constant	−0.6348*** (−4.5868)	−0.2475** (−2.3545)	−0.3005*** (−2.6858)	−0.5358*** (−3.9630)	−0.3779*** (−3.6733)	−0.3332*** (−2.6015)
Observations	1094	1094	1104	1084	1518	670
*R*-squared	0.173	0.174	0.212	0.136	0.121	0.244
Controls	Yes	Yes	Yes	Yes	Yes	Yes
Province fixed effects	Yes	Yes	Yes	Yes	Yes	Yes
Industry fixed effects	Yes	Yes	Yes	Yes	Yes	Yes

This table presents the heterogeneity test results for the impact of ESG performance on the cumulative abnormal return. The dependent variable is CAR in Table 7. We control for the Lev, Roa, Size, Soe, Bate, Top1, Board, Dual, Independ, Intangible, and Tangible. Detailed variable definitions are presented in the Table 1. All variables are winsorised at the 1st and 99th percentiles. *t*-statistics are reported below coefficient estimates and are calculated based on robust standard errors. *, **, and *** denote significance at the 10%, 5%, and 1% level, respectively.

#### Cross-sectional test of public image characteristics

This subsection further investigates whether or not our main results are influenced by corporate image. The concepts of corporate image and trust have obtained special relevance, which significantly influence individual behavior (Rindell et al., 2015). Flavián et al. (2005) argued that the image perceived by consumers makes the factors existing in the transaction visible, thereby reducing the risk perceived by individuals and increasing the possibility of purchase. Compared with firms with bad image, firms with good image are expected to have stronger protection against downside risks (Lee et al., 2022). Consequently, we predict that firms with bad image have a high downside risk during the COVID-19 pandemic, thereby possibly increasing the sensitivity of ESG performance to cumulative abnormal returns.

This study chooses the number of negative reports in online media (Bad_image) measure corporate image. Columns (3) and (4) of Table 7 report that the coefficient of ESG is significantly positive at the 1% level among firms with numerous negative online media reports (firms with Bad_image above the sample median), but it is not statistically significant at a low number of negative online media reports. This finding is consistent with our expectation that firms with bad image face high downside risk during the COVID-19 pandemic, thereby increasing the sensitivity of ESG performance to cumulative abnormal returns.

#### Cross-sectional test of regional characteristics

Lastly, we explore the heterogeneous effect of ESG performance on cumulative abnormal returns for firms with different geographical locations. Shen et al. (2020) noted that by using the region as a criterion, COVID-19 has a major negative influence on the serious-impact regions. After China began its comprehensive campaign against COVID-19, seven provinces, namely, Hubei, Hunan, Henan, Jiangxi, Anhui, Guangdong, and Zhejiang, enforced harsh labor restrictions and the resumption of work. These restrictions have led to a decline in consumption levels and closure of many firms in high-impact areas. By contrast, for cities far from the infected areas, the resumption of operations will be significantly earlier. Fu and Shen (2021) showed that the early resumption of work sends a signal of reduced risk to stakeholders, thereby promoting firms to obtain more investment capital. In summary, we expect that the protection of ESG performance against downside risk during the COVID-19 pandemic is more significant in high-impact regions.

We draw on the previous literature (Shen et al., 2020) in selecting seven provinces (i.e., Hubei, Hunan, Henan, Jiangxi, Anhui, Guangdong, and Zhejiang) as high-impact regions and other provinces as low-impact regions. Columns (5) and (6) of Table 7 show that the coefficient of ESG is significantly positive at the 1% level for firms in high-impact regions, but it is not statistically significant for firms in low-impact regions. This result suggests that ESG practices of firms in high-impact regions play a key role in risk management and reduce stock price volatility during the COVID-19 pandemic.

## Conclusion

During the COVID-19 pandemic, the significant decrease in global equity values reflects strong negative investor sentiments (Broadstock et al., 2021). Hence, we ask if this negative sentiment transfers asymmetrically across the firms, or whether or not ESG performance may be used as a valuable signal for systematically avoiding negative risk during the crisis. However, only a few studies have provided the specific role of ESG performance in this crisis period. Therefore, the goal of this paper is to fill in this research gap.

We use a unique environmental setting and find that ESG performance is positively associated with cumulative abnormal returns around the COVID-19 pandemic and has an asymmetric impact. We contribute to the literature with empirical evidence on the resilience of stocks with high ESG performance during financial crises. This finding is consistent with the view that investors may take ESG performance as a signal of risk mitigation during the crisis. We further find that the reputation and insurance effects are important mechanisms through which ESG performance influences stock price. Moreover, heterogeneous analyses show that ESG effects are considerably pronounced among firms with low human capital, bad image, and in high-impact regions. Overall, we conclude that ESG practices can be used as a risk management tool to enhance share price resilience, particularly in turbulent times.

Our findings are of particular relevance to business managers, investors, and policy makers. For managers, this paper provides empirical evidence supporting ESG investing as a value-enhancing strategy. In addition, we find evidence that ESG practices act as impression and risk management tools to reduce risk downside in turbulent times. Thus, firms should elevate their ESG performance to make them markedly attractive targets in the market to expand their market share. For investors, ESG investments improve the performance of managed portfolios, reduce portfolio risk, and increase returns. Investors should consider ESG factors in their investment decisions to enhance investment returns in turbulent times. Lastly, policy makers should advocate the adoption of ESG practices and encourage companies to disclose information on ESG performance, which are essential for economic sustainability.

Several limitations should be considered in future research. One of the limitations of this study is that the data set only includes large listed firms selected from the China Securities Market and Accounting Research Database. In our opinion, limited resources may play a key role in determining the ESG performance of small firms. Consequently, the inclusion of small- and medium-sized firms may provide different results, which will be left for future research. Furthermore, this study is mainly based on a sample of Chinese listed companies, thereby limiting the generalizability of this research. Lastly, future research could investigate whether or not these results are valid in the context of developed countries or international markets, in which business strategies and ESG disclosures of firms are influenced by the institutional environment.

## Data Availability

Data set used in this study is available from the corresponding author on the reasonable request. Further data is publically available on China Securities Markets and Accounting Research Databases.

## References

[CR1] Al-Awadhi AM, Alsaifi K, Al-Awadhi A, Alhammadi S (2020). Death and contagious infectious diseases: impact of the COVID-19 virus on stock market returns. J Behav Exp Finance.

[CR2] Albuquerque R, Koskinen Y, Yang S, Zhang C (2020) Love in the time of COVID-19: the resiliency of environmental and social stocks. CEPR Discussion Papers 10.1093/rcfs/cfaa011 PMC7454887 40504189

[CR3] Baldini M, Maso LD, Liberatore G, Mazzi F, Terzani S (2018). Role of country- and firm-level determinants in environmental, social, and governance disclosure. J Bus Eth.

[CR4] Broadstock DC, Chan K, Cheng LTW, Wang X (2021). The role of ESG performance during times of financial crisis: evidence from COVID-19 in China. Finance Res Lett.

[CR5] Bushee BJ, Noe CF (2000). Corporate disclosure practices, institutional investors, and stock return volatility. J Account Res.

[CR6] Campbell JY, Mackinlay AC, Lo AW (1998). The econometrics of financial markets. J Empir Finance.

[CR7] Chang Y, Benson K, Faff R (2017). Are excess cash holdings more valuable to firms in times of crisis? Financial constraints and governance matters. Pac-Basin Finance J.

[CR8] Demers E, Hendrikse J, Joos P, Lev B (2021). ESG did not immunize stocks during the COVID-19 crisis, but investments in intangible assets did. J Bus Finance Account.

[CR9] Díaz V, Ibrushi D, Zhao J (2021). Reconsidering systematic factors during the Covid-19 pandemic—the rising importance of ESG. Finance Res Lett.

[CR10] Duuren EV, Plantinga A, Scholtens B (2016). ESG integration and the investment management process: fundamental investing reinvented. J Bus Eth.

[CR11] Erragraguy E, Revelli C (2015). Should Islamic investors consider SRI criteria in their investment strategies. Finance Res Lett.

[CR12] Fahlenbrach R, Rageth K, Stulz RM (2020) How valuable is financial flexibility when revenue stops? Evidence from the COVID-19 crisis. Working Paper Series

[CR13] Fama E, Fisher L, Jensen M, Roll R (1969). The adjustment of stock prices to new information. Int Econ Rev.

[CR14] Fernando CS, May AD, Megginson WL (2012). The value of investment banking relationships: evidence from the collapse of lehman brothers. J Finance.

[CR15] Flavián C, Guinalíu M, Torres E (2005). The influence of corporate image on consumer trust: a comparative analysis in traditional versus internet banking. Internet Res.

[CR16] Friedman M (2007) The social responsibility of business is to increase its profits. In: Zimmerli WCh, Holzinger M, Koordination CSR und Nachhaltigkeit (eds). Corporate ethics and corporate governance. Springer, pp. 173–178

[CR17] Fu M, Shen H (2021). COVID-19 and corporate performance in the energy industry. Energy Res Lett.

[CR18] Galbreath J (2013). ESG in focus: the Australian evidence. J Bus Eth.

[CR19] Ghosh D, Olsen L (2009). Environmental uncertainty and managers’ use of discretionary accruals. Account Organ Soc.

[CR20] Hartzmark SM, Sussman AB (2019). Do investors value sustainability? A natural experiment examining ranking and fund flows. J Finance.

[CR21] Henke H-M (2016). The effect of social screening on bond mutual fund performance. J Bank Finance.

[CR22] Hoepner AGF, Oikonomou I, Sautner Z, Starks LT, Zhou X (2021) ESG shareholder engagement and downside risk. Soc Sci Res Netw. Finance Working Paper No. 671/2020.

[CR23] Jagannathan R, Ravikumar A, Sammon M (2017) Environmental, social, and governance criteria: why investors are paying attention. National Bureau of Economic Research

[CR24] Kanas A (2005). Pure contagion effects in international banking: the case of BCCI’s failure. J Appl Econ.

[CR25] Lee S, Lee D, Hong C, Park M-H (2022). Performance of socially responsible firms during the COVID-19 crisis and trading behavior by investor type: evidence from the Korean stock market. Finance Res Lett.

[CR26] Li K, Liu X, Mai F, Zhang T (2021). The role of corporate culture in bad times: evidence from the COVID-19 pandemic. J Financ Quant Anal.

[CR27] Lokuwaduge CSDS, Heenetigala K (2017). Integrating environmental, social and governance (ESG) disclosure for a sustainable development: an Australian study. Bus Strategy Environ.

[CR28] Manabe T, Nakagawa K (2022). The value of reputation capital during the COVID-19 crisis: evidence from Japan. Finance Res Lett.

[CR29] Mǎnescu C (2011). Stock returns in relation to environmental, social and governance performance: mispricing or compensation for risk?. Sustain Dev.

[CR30] McWilliams A, Siegel D (1997). Event studies in management research: theoretical and empirical issues. Acad Manag J.

[CR31] Miyajima H, Yafeh Y (2007). Japan’s banking crisis: an event-study perspective. J Bank Finance.

[CR32] Nejati M, Shah Bin A, Shahbudin, Bin Amran A (2010). Sustainable development: a competitive advantage or a threat?. Bus Strategy Ser.

[CR33] Pandey DK, Kumari V (2021). Event study on the reaction of the developed and emerging stock markets to the 2019-nCoV outbreak. Int Rev Econ Finance.

[CR34] Pedersen LH, Fitzgibbons S, Pomorski L (2021). Responsible investing: the ESG-efficient frontier *. J Financ Econ.

[CR36] Ren Z, Zhang X, Zhang Z (2021). New evidence on COVID-19 and firm performance. Econ Anal Policy.

[CR37] Renneboog L, Ter Horst J, Zhang C (2008). Socially responsible investments: institutional aspects, performance, and investor behavior. J Bank Finance.

[CR38] Rindell A, Santos FP, de Lima AP (2015). Two sides of a coin: connecting corporate brand heritage to consumers’ corporate image heritage. J Brand Manag.

[CR39] Sassen R, Hinze A-K, Hardeck I (2016). Impact of ESG factors on firm risk in Europe. J Bus Econ.

[CR40] Shen H, Fu M, Pan H, Yu Z, Chen Y (2020). The impact of the COVID-19 pandemic on firm performance. Emerg Markets Finance Trade.

[CR41] Tang C-H, Chin C-Y, Lee Y-H (2021). Coronavirus disease outbreak and supply chain disruption: evidence from Taiwanese firms in China. Res Int Bus Finance.

[CR42] Wang L, Dai Y, Kong D (2021). Air pollution and employee treatment. J Corporate Finance.

[CR43] Xie J, Nozawa W, Yagi M, Fujii H, Managi S (2019). Do environmental, social, and governance activities improve corporate financial performance?. Bus Strategy Environ.

[CR35] Yu EP, Luu BV (2021). International variations in ESG disclosure—do cross-listed companies care more. Int Rev Financ Anal.

[CR44] Zhang P, Gao J, Li X (2021). Stock liquidity and firm value in the time of COVID-19 pandemic. Emerg Mark Finance Trade.

